# Image Tracking Study on Courtship Behavior of Drosophila

**DOI:** 10.1371/journal.pone.0034784

**Published:** 2012-04-04

**Authors:** Hung-Yin Tsai, Yen-Wen Huang

**Affiliations:** Department of Power Mechanical Engineering, National Tsing Hua University, Hsinchu, Taiwan, Republic of China; Imperial College London, United Kingdom

## Abstract

**Background:**

In recent years, there have been extensive studies aimed at decoding the DNA. Identifying the genetic cause of specific changes in a simple organism like Drosophila may help scientists recognize how multiple gene interactions may make some people more susceptible to heart disease or cancer. Investigators have devised experiments to observe changes in the gene networks in mutant Drosophila that responds differently to light, or have lower or higher locomotor activity. However, these studies focused on the behavior of the individual fly or on pair-wise interactions in the study of aggression or courtship. The behavior of these activities has been captured on film and inspected by a well-trained researcher after repeatedly watching the recorded film. Some studies also focused on ways to reduce the inspection time and increase the accuracy of the behavior experiment.

**Methodology:**

In this study, the behavior of drosophila during courtship was analyzed automatically by machine vision. We investigated the position and behavior discrimination during courtship using the captured images. Identification of the characteristics of drosophila, including sex, size, heading direction, and wing angles, can be computed using image analysis techniques that employ the Gaussian mixture model. The behavior of multiple drosophilae can also be analyzed simultaneously using the motion-prediction model and the variation constraint of heading direction.

**Conclusions:**

The overlapped fruit flies can be identified based on the relationship between body centers. Moreover, the behaviors and profiles can be correctly recognized by image processing based on the constraints of the wing angle and the size of the body. Therefore, the behavior of the male fruit flies can be discriminated when two or three fruit flies form a close cluster. In this study, the courtship behavior, including wing songs and attempts, can currently be distinguished with accuracies of 95.8% and 90%, respectively.

## Introduction

The sequence of human genomes has been revealed in recent years. To understand the relationship between human genes and behavior, biologists have studied the behavior of Drosophila, whose genes have a 60% similarity to those of human beings. The relationship can be determined by observing the behavior of fruit flies with different mutations [1]–[2]. Currently, the activity behavior is captured on film and inspected by a well-trained researcher. The features of this behavior are manually noted after repeatedly watching the recorded film. Many studies have focused on ways to reduce the inspection time and increase the accuracy of the behavior experiments that employ machine vision. The locomotion of a single fruit fly or a group of flies in different circumstances has been analyzed in related studies [3]–[6]. Branson et al. proposed the analysis of probability density and motion prediction to track multiple fruit flies without wings and analyze the motions of flies with different sexes [7]. Many studies have also focused on the behavior responses using different stimuli [8]–[9]. Biologists have also focused on innate behavior such as courtship. Dankert et al. have identified the orientation, size, and wing angles of a fruit fly using the Gaussian mixture model [10]. Moreover, the courtship behavior of a couple can be completely discriminated based on their locomotion analysis.

In this study, the features of the Drosophila's profile such as size, orientation, and wing angles can be analyzed employing the Gaussian mixture model. When many flies are in a cluster, the profile and locomotion of a single fruit fly can be recognized using the motion-prediction model. The courtship behavior, including wing songs and attempts, can be discriminated by setting up the range for profile identification and locomotion.

## Methods

### Equipment Design

The experiment was conducted in a circle glass container of 20-mm inner diameter and 40-mm outer diameter. The heights of the inner and outer walls are 3 mm and 30 mm, respectively. The area is covered by a piece of glass coated with a hydrophobic agent to prevent the fruit flies from climbing up the wall. The small size of the inner diameter is designed to increase mutual activity among the flies and the low wall prevents the fruit flies from jumping. The light passing through the outer wall can even simplify the inspection because of the coarse surface design. The light source is a line-shaped LED array filtered by the diffuser.

### Identification of Fruit Fly Appearance

**Figure 1:**
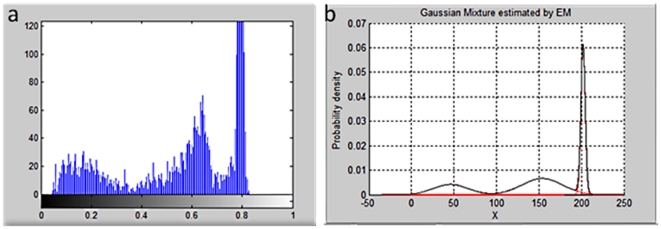
The left image is the histogram of intensity in which the vertical axis is the quantity and the horizontal axis is the intensity. The right image is the distribution of the probability density in which the vertical axis is the probability density and the horizontal axis is the intensity.

**Figure 2:**
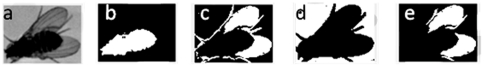
(a) The original gray image. (b), (c), and (d) are the patch of the body, wings, and background, respectively. (e) The clear wing patch after morphological operation.

The clusters in the foreground are extracted by background subtraction and are analyzed using the Gaussian mixture model. The histogram intensity can be fitted by three different Gaussian distributions as shown in Fig. 1. These three parts can be respectively represented as the abdomen, wings of a fruit fly, and the background based on the average intensity of each part from dark to light as shown in Fig. 2.

**Figure 3:**
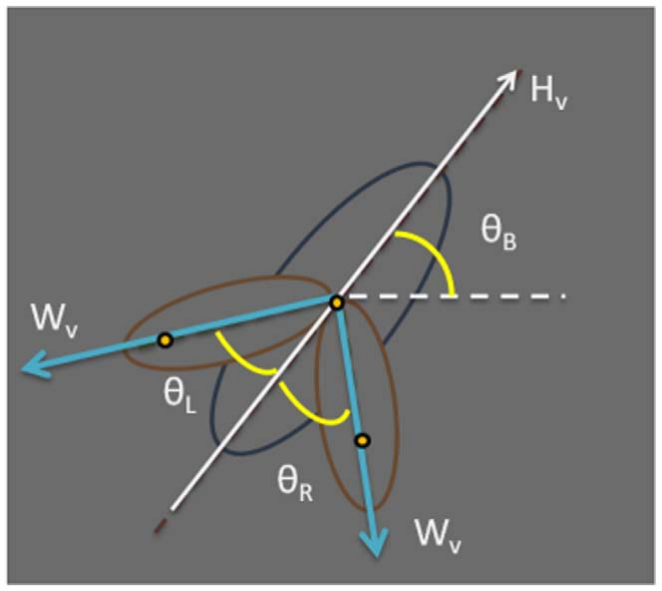
The schematic figure of the profile of a fruit fly. The blue ellipse is the fruit fly body, the orange ellipse represents wings, the white arrow represents the heading direction of the fruit fly, θ_B_ is the angle between the heading direction H_v_ (white arrow) and the lateral axis, and W_v_ is the directional vector of the wing center to the body center. The wing angles are defined as the acute angles between the blue and white lines.

When the three parts are recognized by the Gaussian distributions, the profile of the body and wings, including the area, center of mass, position, and direction of each fruit fly can thereby be identified. The wing angles θ_L_ and θ_R_ are defined by the acute angle between the connection of body and wing centers and the direction of the body as shown in Fig. 3.

Since the maximum wing angle is not larger than 90°, the angle between the vector H_v_ and W_v_ must exceed 90°. The heading direction H_v_ can therefore be identified based on the above constraint. In addition, the variation of the heading direction is continuous. Therefore, the direction at time t can be modified by comparing the difference of the directions at time t and at time t-1. Thus, the variation of the directions within the 0.1-sec period between continuous images is confined to within 90°. If the variation is larger than 90°, the heading direction H_v_ is adjusted to be reversal. Otherwise, it remains the same.

### Tracking of Fruit Flies

**Figure 4:**
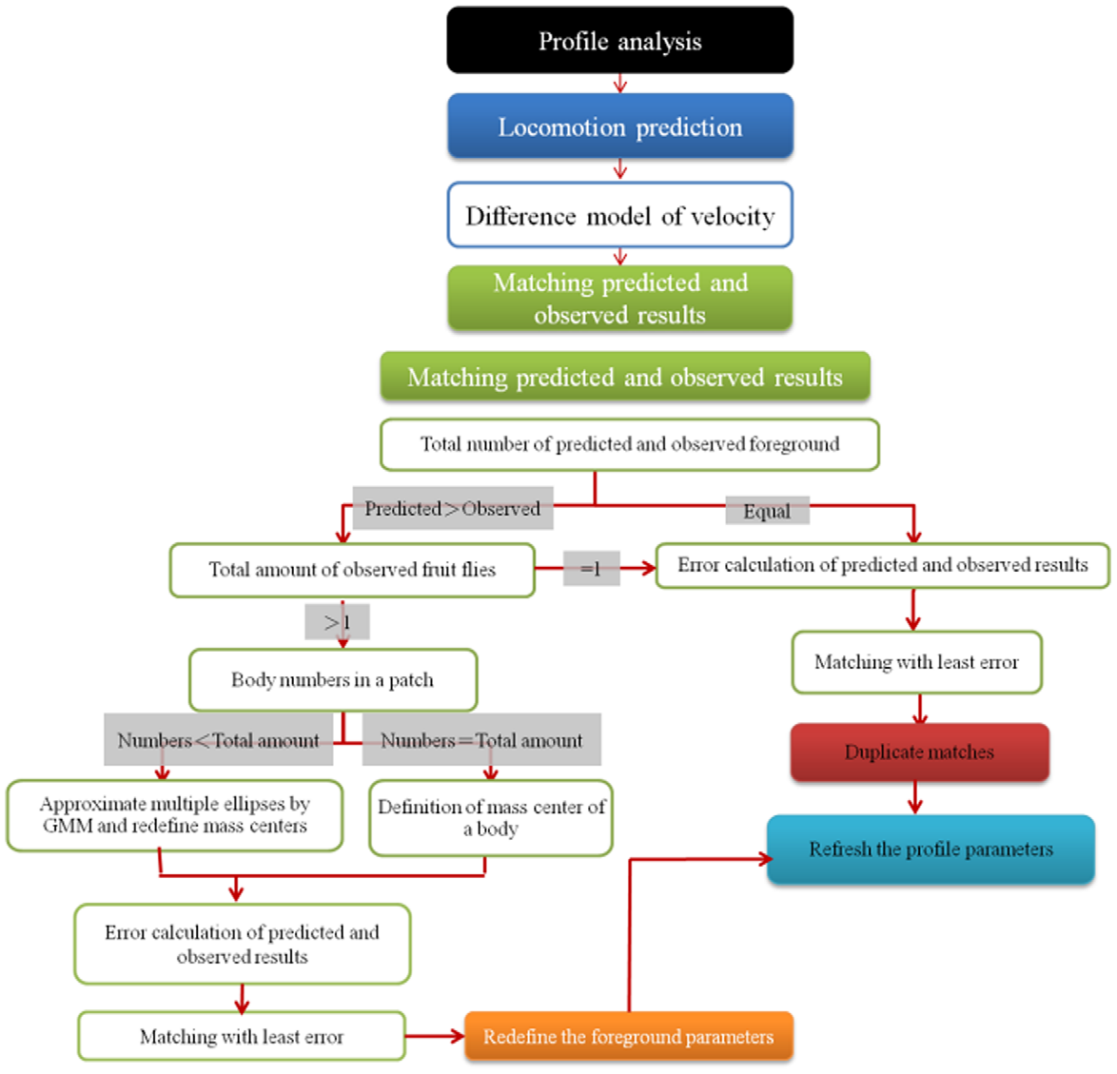
Flow chart showing the tracking technique and the discrimination of multiple fruit flies.

The technique used for the tracking and discrimination of multiple fruit flies in this study comprises the following three parts: motion prediction, matching of the predicted and observed positions, and the refreshed profile of the fruit fly. Fig. 4 shows the flow chart of the tracking process. The position of each fruit fly is predicted based on the analyzed profile and the motion-prediction model. The result is matched with that of the observed fruit fly. In the case where the position cannot be correctly predicted because of the overlapping of multiple fruit flies, we analyze these clusters, define the new center of each fruit fly, and then relate the new center to the observed position of each fruit fly. If there are duplicate matches, the variations of distance and orientation at the points of time t and t-1 will be used to find the updated optimal match. Finally, the correct profile of each fruit fly obtained from the overlapping of multiple fruit flies is renewed and the refreshed result is employed for subsequent track prediction.

### Difference Model of Velocity

The match that is determined for each fruit fly in different frames is based on the minimum error between the predicted and observed positions of fruit flies. The prediction is according to the difference model of the velocity. We assume that the velocity of a fruit fly moving from time t-1 to time t is equal to that from time t-2 to time t-1. The position of the fruit fly is x_t-1_ at time t-1 and x_t-2_ at time t-2. The predicted position x^pr^ at time t can then be calculated by the difference model of the velocity as shown in Eq. (1).
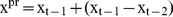
(1)


The predicted and observed positions are expressed as 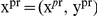 and 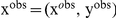 respectively. Therefore, the error at time t can be presented in Eq. (2) as:
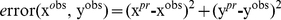
(2)


### Matching

**Figure 5:**
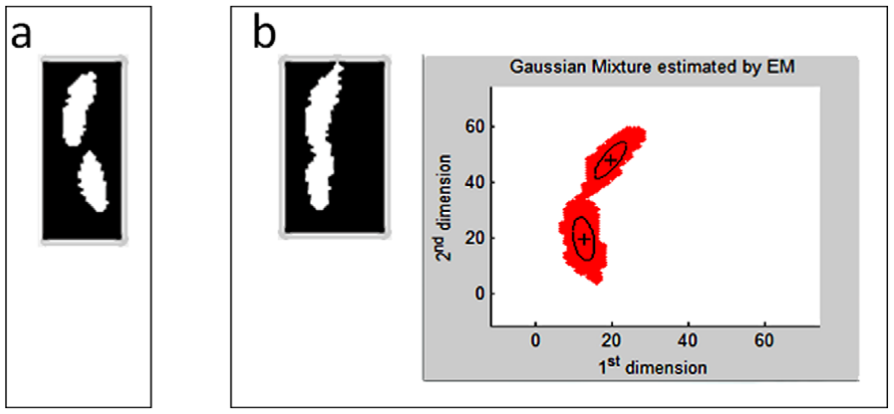
Overlapping image patch and body centers determined by the Gaussian mixture fitting.

The correct match is determined by the least error of the predicted and observed positions. The error is calculated based on the center of the body when the fruit flies are separated. When the fruit flies overlap in the foreground cluster, the body parts are fitted by the Gaussian mixture model and the body centers are renewed for further matching as shown in Fig. 5.

### Discrimination of Courtship Behavior of Fruit Flies

**Figure 6:**
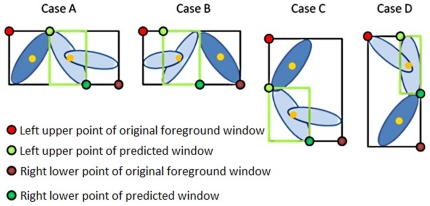
The schematic for determining the patch boundaries for two overlapping fruit flies (one male and one female).

The profiles of the overlapping fruit flies must be refreshed after matching. The images of the new foreground clusters are divided into two parts having two and three fruit flies, respectively. It is also assumed that the wings of the female fruit fly do not open during courtship. In the case of two overlapping fruit flies including one male and one female fruit fly, the region of the cluster at time t is determined based on the one used at time t-1 if the fruit fly is female. If the fruit fly is a male whose wing angle has the features associated with courtship behavior, the new cluster of two flies is considered to be refined as shown in Fig. 6. The dark blue ellipse is the body of a female fruit fly and the big light-blue ellipse is the body of a male fruit fly, with the small light-blue ellipse as a wing of the male fruit fly.

According to the horizontal and vertical distances between the centers of fruit flies in the rectangle patch, the corresponding positions of the male and female fruit flies were identified. Some boundaries of the patch used at time t-1(the light green window) can be extended to the boundaries of the black window, which are the patch boundaries of the overlapping fruit flies. This will enable us to find the wing position of the male fruit fly. For example, if the female fruit fly is to the right of the male, the right boundary of the extracted patch of the male fruit fly remains the same, and the other three boundaries are extended to the black ones as with Case B in Fig. 6.

**Figure 7:**
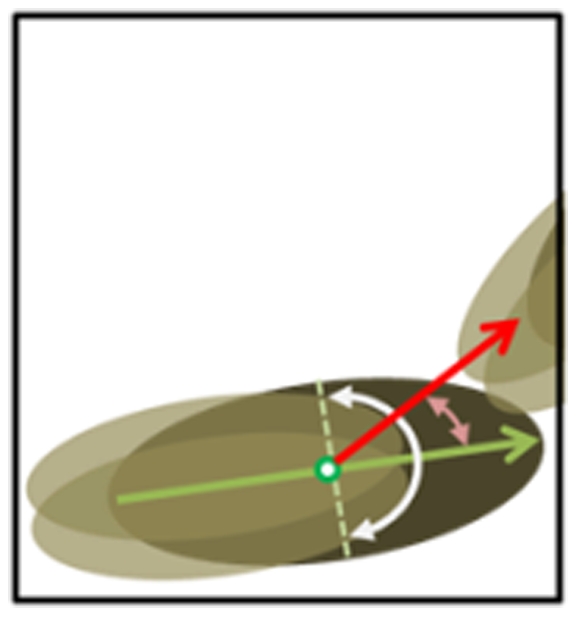
The schematic for determining the body and wing region of the target fruit fly.

In the case of three overlapping fruit flies (two males and one female), the boundary of the cluster is determined based on the constraints of the wing angles and the body size. Body parts not belonging to the target fruit fly are eliminated if they are outside the region obtained by a circle that is centered at the mass center of the target fruit fly and which has a diameter that is the length of the target. As shown in Fig. 7, the black window is the region of the foreground. The green arrow points in the heading direction of the fruit fly and the white angle range shows the incorrect direction in which the wings are pointed. Based on the wing angle constraints (less than 90°), the wing angle, such as the red arrow, is not practical and the wing parts are eliminated.

In this study, we focus on differentiating between the following two kinds of courtship behavior. First, a male fruit fly will move a single wing with a specific frequency as a sort of “love song” or “wing song” with the purpose being to attract a female. The other is that a male fruit fly will approach and lick the tail of a female or try to copulate. The feature of courtship is determined based on the parameters of the profile and the locomotion of fruit flies, as shown in Table 1. Therefore, the parameters used to recognize wing song behavior are defined in Table 2.

**Table 1:** Parameters of the profile and locomotion of fruit flies.

Parameters	Definition
Position p	x, y coordinates of the body center of a fruit fly
Displacement s	Deviation of body centers of a fruit fly at images between time t and time t-1
Velocity v	Displacement divided by the time period
Heading Direction θ_B_	Longitudinal axis of a fruit fly body and heading to the direction on the opposite of the wings based on the body center
Wing Angle θ_L_, θ_R_	Wing angle between the longitudinal axis of a fruit fly body and the left or the right wing
Angle ψ_12_	Angle between the heading direction of a male fruit fly and connection of body centers of the male and a female fruit flies
Angle γ_12_	Angle between the heading direction of a male fruit fly and connection of the body center of the male fruit fly and the abdomen (genital) of a female fruit fly
Δc-c	Distance between body centers of different fruit flies
Δc-a	Distance between the body center of a male fruit fly and the abdomen (genital) of a female fruit fly

**Table 2:** Parameters used to recognize wing song behavior.

Parameters	Fruit Fly 1 or 2	Time	Duration		Unit
			min	max	
Δ_c-c_	1–2	t	0	4	mm
ψ_12_	1–2	t	0	45	°
Wing Angleθ_L_ or θ_R_	1	t	30	95	°

Dankert et al., 2009, used 60° as a threshold [10], while we used 30°. For a male fruit fly, we used the wing-angle range of 30° to 95° to indicate courtship behavior in order to avoid missing frames with large wing angles when the frame rate is relatively slow. The upper bound of 95°, which is slightly larger than the constraint of 90°, is set to reduce the effect of position errors caused by the deviation of body and wing centers.

When a male fruit fly wants to lick or touch the tail of a female, the distance and relative velocity between the two fruit flies will decrease. The ranges of related parameters are defined in Table 3 to differentiate between these features.

**Table 3:** Parameters for differentiating among the attempted behaviors.

Parameters	Fruit Fly 1or 2	Time	Duration		Unit
C_1_A_2_<C_2_A_1_					
			min	max	
Δ_c-c_	1–2	t	0	4	mm
Δ_c-a_	1–2	t	0	2	mm
γ_12_	1–2	t	0	45	°
Velocity v	1,2	t	0	5	mm/s

The range of the velocity is determined based on the result obtained in the study done by Branson et al., 2009, in which the walking speed is defined as being larger than 5 mm/s [7]. Therefore, the upper bound of the velocity is set to 5 mm/s when courtship behavior occurs.

## Results and Discussion

**Figure 8:**
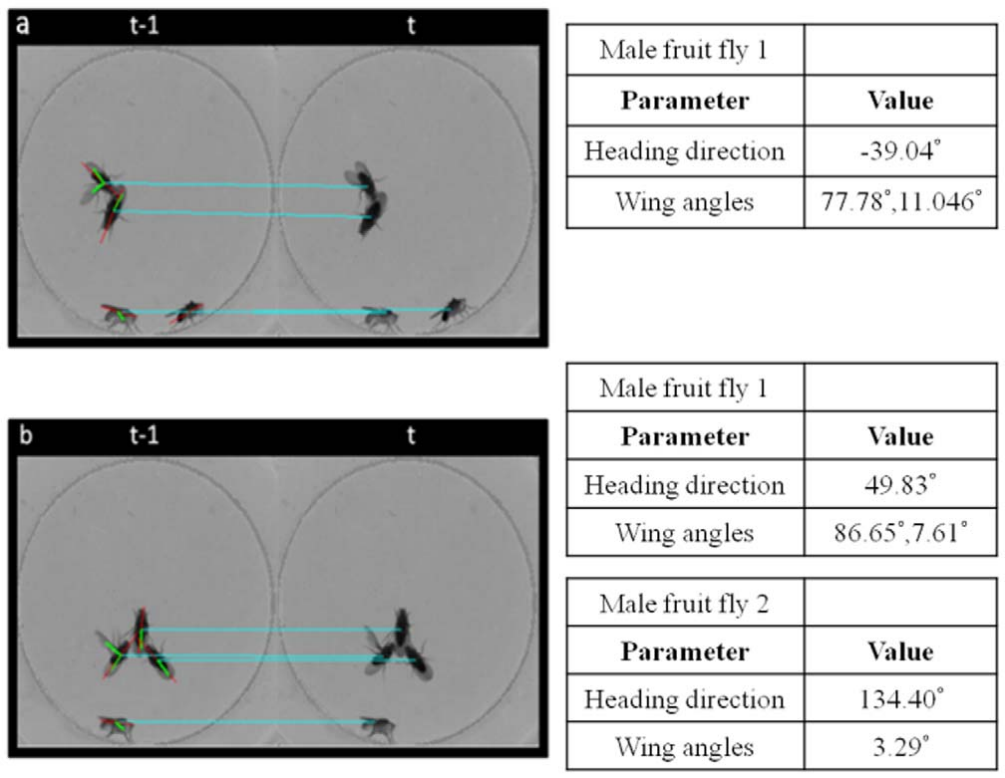
The analyzed result of the heading direction and wing angles of multiple overlapping flies.

The profile and motion of overlapping flies are recognized concerning the positions of the centers of the body and wings, as well as the constraints of body size and wing angle. The heading direction and wing angles are labeled for the case of two or three overlapping flies existing in a cluster of two males and two females, as shown in Fig. 8. The source code and instruction of operation can also be obtained in File S1 and File S2. A sample movie can also be shown in Movie S1.

**Figure 9:**
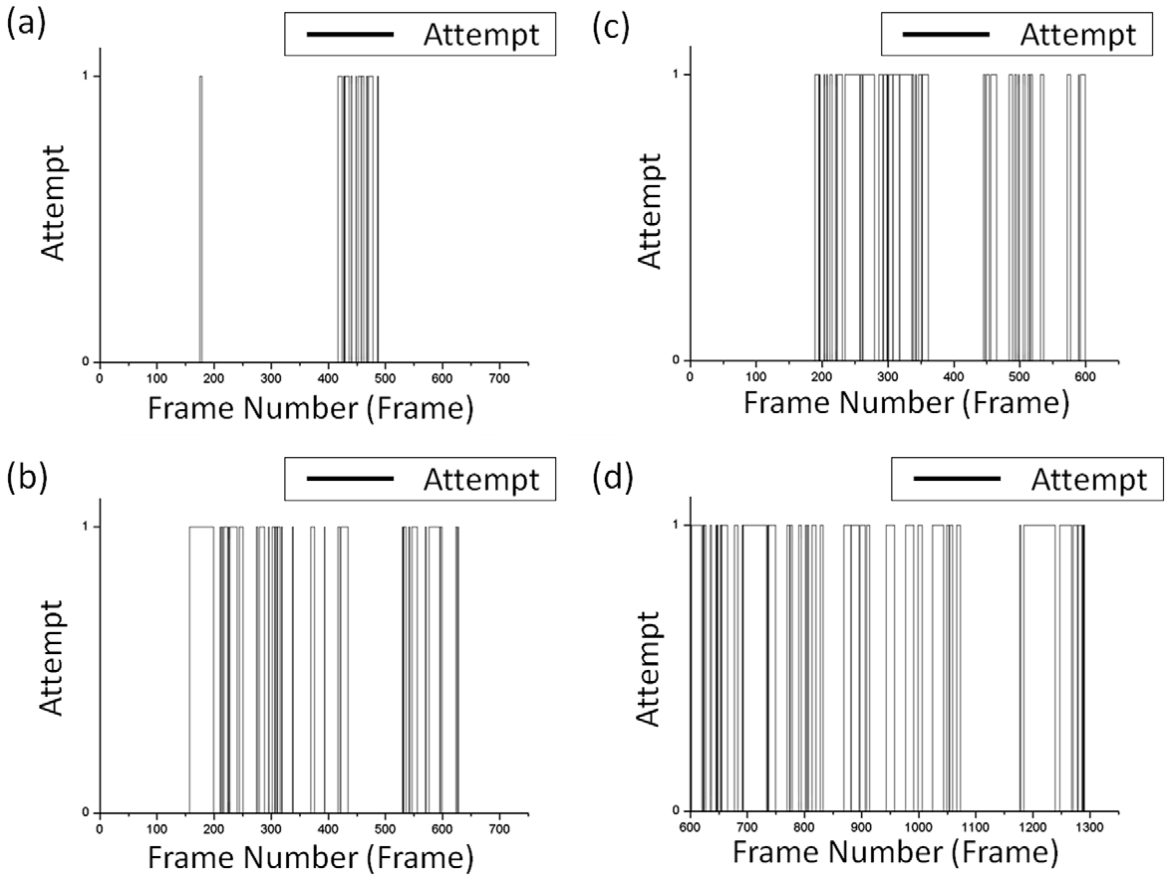
The detected result of the discrimination of attempt behavior. (a) and (b) show the first sequential images. (c) and (d) show the second sequential images.

To identify courtship behavior, the resolution of the image is 250×250 and the frame rate is 5 Hz. The continuous images taken at intervals of two and four minutes are analyzed. Of the 1750 images that were analyzed, it took an average of 0.73 s to analyze each frame. In the analyzed result, courtship features are indicated by 1 and other features are represented by 0, as shown in Figs. 9–11.

**Figure 10 pone-0034784-g010:**
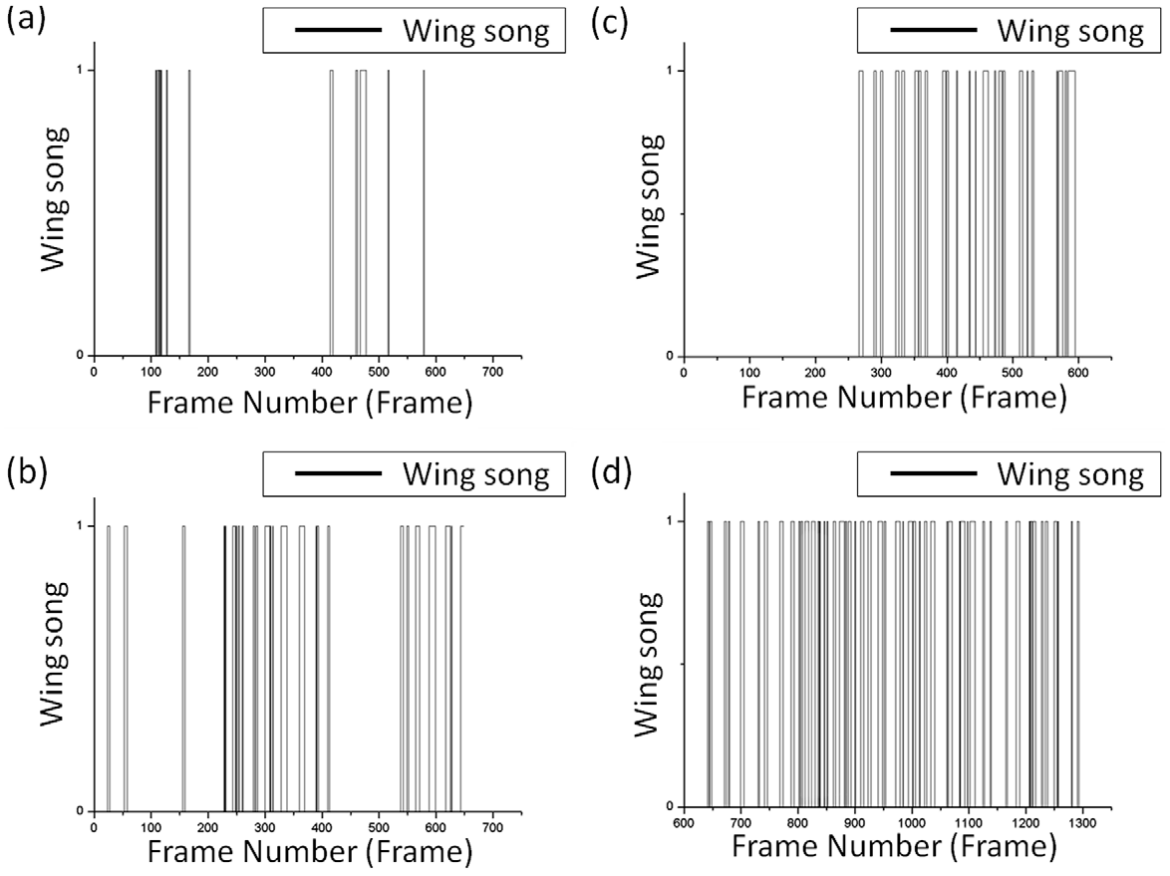
The detected result for the discrimination of wing song behavior. (a) and (b) show the first sequential images. (c) and (d) show the second sequential images.

**Figure 11:**
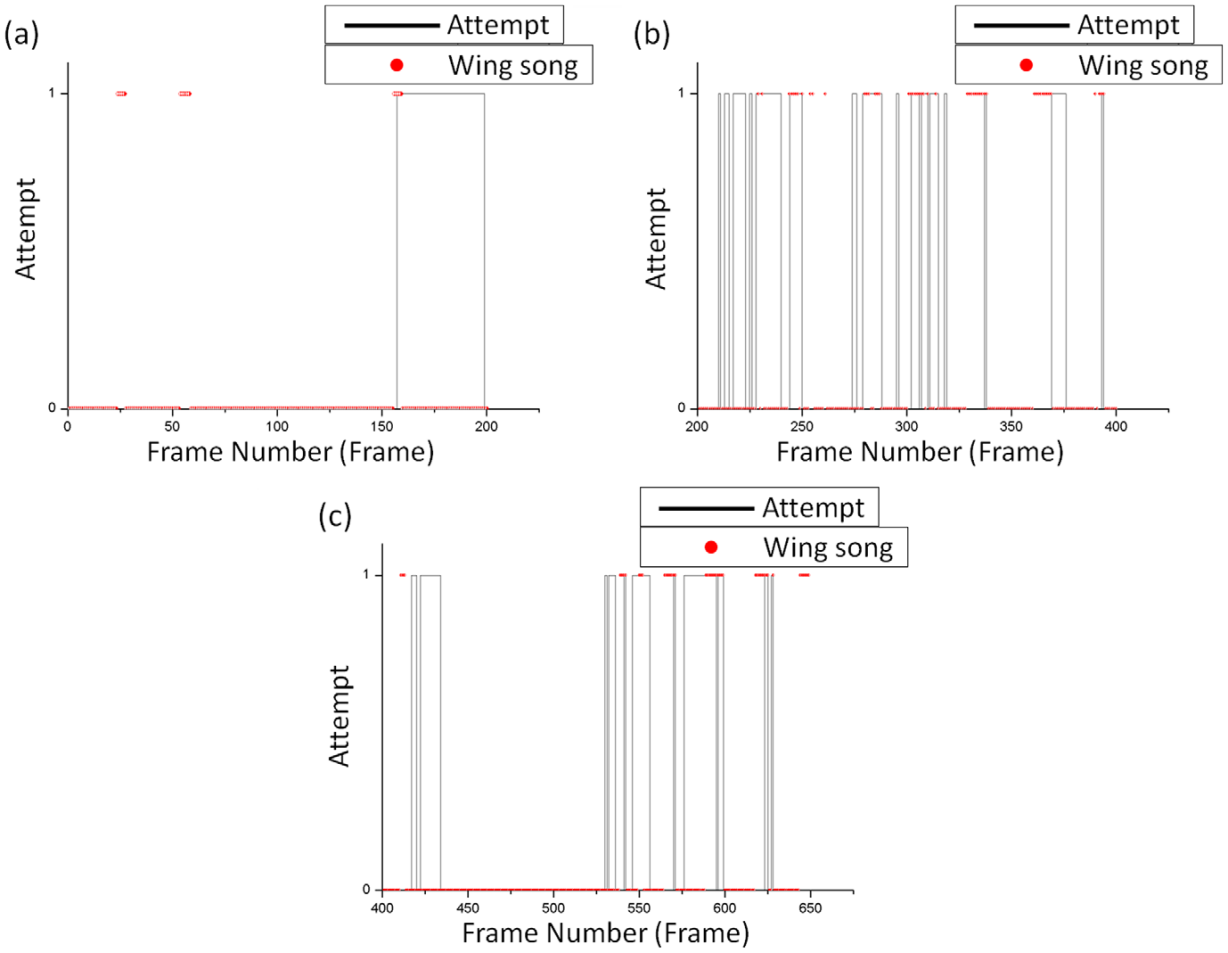
Comparison of attempt and wing-song behavior. The red dots represent the wing song behavior and the black line represents the attempt behavior.

The behavior of a male fruit fly as it approaches the tail of a female fruit fly is defined as “attempt.” Fig. 9 shows the detected result of the attempted behavior between continuous images. The distribution shows that each attempt spans about 100 images and lasts for about 20 seconds.

The wing-song behavior of a male fruit fly fluttering a single wing is shown in Fig. 10. This event spans about 5 to 10 images and lasts for about 1 to 2 seconds, and each wing-song is repeated after a short period of rest. Moreover, a male fruit fly will not flutter its wing to suggest courtship while another male is doing the same.

The results used to detect the attempt behavior and the wing song were compared in order to determine if there exists any behavioral sequence such as that shown in Fig. 11. At first, there was a wing song followed by an attempt, as shown in Fig. 11 (a). After that, there was no obvious behavioral sequence since the two kinds of behavior happened occasionally, as shown in Fig. 11 (b) and (c). However, wing-song events were often detected before or after the attempt event. The male tried very hard to attract and copulate with the female fruit fly even when the female refused, and this was observed on the video. The attempt events therefore occurred frequently and were accompanied by wing-song events at frames 200 to 400.

The correct discrimination accuracy is defined as the ratio of the number of the correctly identified images to that of the total images as expressed in Eq. (3).
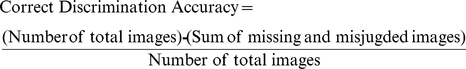
(3)


Of the 649 images in the first sequential images obtained, the number of detected attempt behaviors for the first and second male fruit flies are 42 and 169, respectively. The correct discrimination accuracies were 99.6% and 95.99%, respectively. Of the 1293 images in the second sequential images, the detected number of male fruit flies with high attempt behavior is 505. The correct discrimination accuracy is 89.8%. The incorrect detections often occurred at the beginning and end of a long period of an event, and thus these kinds of errors rarely affect the detected results.

Of the 649 images in the first sequential images, the number of occurrences of the wing-song behavior was 25 and 106 times, respectively, and the correct discrimination accuracies were both 99.8%. The misjudged cases occurred when the male fruit fly that was actually on the side wall was recognized as the rear of a body. Of the 1293 images, the detected number of male fruit flies with high wing-song behavior is 287, and the correct discrimination accuracy is 95.8%. The cases with incorrect detections occurred at the beginning and end of the wing-song behavior because of errors in determining the small angles of overlapping wings. However, these kinds of errors rarely affected the detected results.

### Conclusion

In this study, we propose a technique that can be used to track and differentiate among different behaviors of Drosophila to overcome the difficulty of identifying the courtship behavior of clusters of multiple flies. This technique was developed using image processing. The overlapping flies can be recognized based on the relative positions of the body centers and the fitting methods of the Gaussian mixture model. Then the profile can be analyzed by the constraints of body size and wing angle. Courtship behavior involving wing songs and attempt events can be differentiated from among the sequential images even in cases with multiple overlapping flies. Currently, the correct discrimination accuracies for the analysis of the wing songs and attempt behavior are 95% and 90%, respectively.

## Supporting Information

File S1 Code for paper.
(RAR)

File S2 Instruction of operation.
(DOC)

Movie S1 Sample movie.
(AVI)
