# Supplementary material for: Image Tracking Study on Courtship Behavior of Drosophila
Source: PLoS One. 2012 Apr 4;7(4):e34784. doi: 10.1371/journal.pone.0034784 (PMC3319603; doi:10.1371/journal.pone.0034784)
Supplement: File S2 — Instruction of operation. (DOC) [file pone.0034784.s002.doc]

Execution of the program

1. Put all the “function” of MATLAB into a same folder (as the attached “code for paper”).
2. Execute MATLAB software. Set the path of Current Dictionary to the folder mentioned above and open the file Id_matching_main.m.
3. Set the file name of the resulted movie at the line 6 of Id_matching_main.m. The input command can be aviobj = avifile('Final_result1D.avi') if the output name is Final_result1D.
4. Put the prepared background image into the same folder of all the “function” and change the file name at the line 23 of Id_matching_main.m. The input command can be im1back=imread('ccfu_c_back.bmp') if the name of the background image is ccfu_c_back.bmp.
5. Change the file name at the line 30 of Id_matching_main.m and set the path of the original images. The input command can be path1=['D:\test_pics\origin\exp_photos\'] if the path is <D:\test_pics\origin\exp_photos\>.
6. Execute Id_matching_main.m.

Extraction of the output data

1. The parameters including body center, wing center, orientation, heading direction, wing angle, and body condition (Back or Side to screen) are recorded in the structure titled parameter_file. As shown in Fig. 1, the first level is a 1xn structure, in which n means different frames. The second level is related to the information of this frame as shown in Fig. 2. The parameters of each fly in the frame can be checked as the label titles static_parameter as shown in Fig. 3.

Fig. 1

Fig.2

Fig.3

1. The data about the moment of the attempt behavior were recorded in the matrix titled Check_Trace_Condition. The columns represent different flies and the rows represent different frames. The digits 1 and 0 in the matrix mean the behavior happened and un-happened, respectively.
2. The data about the distance C1A2 when the attempt behavior took place were recorded in the matrix titled Check_Lick_dis_Final.
3. The data about the velocity when the attempt behavior took place were recorded in the matrix titled Check_Lick _Final.
4. The data about the moment of the wing song behavior were recorded in the matrix titled Check_Wing_Song. The columns represent different flies and the rows represent different frames. The non-zero elements in the matrix represent the behavior happened at that frame.
5. The data about the angle ψ_12_ were recorded in the matrix titled Check_WS_Angle_CCH.
6. The data about the velocity when the wing song behavior took place were recorded in the matrix titled Check_Chasing_Velocity.
